# The Effect of Ketamine on Posttonsillectomy Pain in Children: A Clinical Trial 

**Published:** 2012

**Authors:** Akbar Pirzadeh, Mohammad-Ali Mohammadi, Sooreh Allaf-Akbari, Masood Entezariasl

**Affiliations:** 1 *Department of otorhinolaryngology, Ardabil University of Medical Sciences, Ardabil, Iran*; 2 *Instructor of nursing, Ardabil University of Medical Sciences, Ardabil, Iran*; 3 *Department of anaesthesiology, Ardabil University of Medical Sciences, Ardabil, Iran*

**Keywords:** Children, Ketamine, Postoperative pain, Tonsillectomy

## Abstract

**Introduction:**

Tonsillectomy is one of the most common surgical operations and has such complications as pain, hemorrhage and laryngospasm. Pain management is of vital importance in order to reduce the suffering and restlessness in children having undergone tonsillectomy. Different studies differ in their findings as to the use of ketamine for postoperative analgesia. The aim of this study was to investigate the effect of peritonsillar injection of ketamine preoperatively on postoperative pain relief.

**Materials and Methods:**

This was a randomized controlled trial (RCT) on sixty 3-12-year-old children. Children were randomly assigned to the intervention and control groups. Peritonsillar injection consisted of 1 mg/kg ketamine in the intervention group and of normal saline in the control group. An injection of 1 cc was administered on each side five minutes prior to tonsillectomy. Pain assessment was performed using the self-report Oucher Scale and CHEOPS (Children's Hospital of Eastern Ontario Pain Scale) and sedative state assessment was performed using the Wilson Sedation Scale. Pain, medication and complications were studied for 24 hours. Data analysis was performed using chi-squared test and t-test.

**Results:**

The ketamine group had a lower pain score compared with the control group (1.40±1.003 compared with 1.53±1.074). The average pain was less in the control group two hours after the surgery. The difference was statistically significant. There was no difference between the two groups in terms of nausea and vomiting incidence.

**Conclusion:**

The peritonsillar injection of ketamine five minutes prior to the surgery reduces the post-tonsillectomy pain without causing any complications.

## Introduction

Inflammatory and infectious diseases of the throat, tonsils and adenoids account for a large portion of children’s diseases and healthcare costs. In most cases, these diseases lead to either tonsillectomy or adenectomy, both common child surgeries (1). Tonsillectomy is a common and painful procedure in children. Post-tonsillectomy problems include pain, hemorrhage, laryngospasm, airway obstruction, nausea, vomiting and aspiration (2). 

Several analgesics are used for post-tonsillectomy pain relief, such as narcotics, NSAIDs and local anesthetics. Nonetheless, narcotics can reduce the muscular tone of the upper airway, weaken the cough reflex, and cause respiratory depression, nausea and vomiting after the surgery. The positive effects of preoperative analgesia in the treatment of postoperative pain have been shown in several studies (3, 4). With its antagonistic effects on NMDA (N-methyl-D-aspartate) receptors, ketamine can prevent or reverse neural sensitivity to pain impulses, hence reducing postoperative pain (5). Despite the positive effects of preoperative ketamine analgesia on postoperative pain, some studies (4) show contradictory results as to the effects of IV ketamine (0.5 mg/kg) on post-tonsillectomy pain (6). Dal et al showed that low doses of IV ketamine in peritonsillar injections was effective on postoperative pain relief and had no complications. However, one of the limitations of this study was the lack of a control group (to receive a placebo) in the peritonsillar injections (2).

In several studies, normal saline was used preoperatively in the placebo group to investigate the postoperative effects of the analgesia of other drugs in children undergoing tonsillectomy. Mollier et al showed that postoperative pain in the preoperative peritonsillar injection with bupivacaine was less compared with the control (placebo) group injected with normal saline (7).

In their study, Dal et al (2) not only had no control group for the comparison of the effects of preoperative peritonsillar injection of ketamine on children’s postoperative pain but also did not determine the effective dose of injection. Also, high doses of ketamine in peritonsillar injections cause postoperative analgesia because of its systemic absorption. Therefore, the present triple-blind RTC study with a control (placebo) group was performed to investigate the effects of preoperative analgesia with ketamine 1 mg/kg, in peritonsillar injections, on postoperative pain.

## Materials and Methods

This is a triple-blind RCT study; the patients, the ENT specialist performing the surgery, the anesthesiologist administering the anesthetic and the anesthesiology assistant (MSc) recording the pain and restlessness levels and the postoperative results did not know the patients’ assignment to the two groups or the medication used. The data analyst did not know which patient had received ketamine and which normal saline, either. The statistical population of the sample was the 3-12-year-old tonsillectomy candidates at the Imam Khomeini Hospital, Ardabil, Iran. The sample size with 80% power of study and considering 20% flaw was calculated to be 30. 

Patients’ assignment to normal saline or ketamine groups was random.

After acquiring permission from the Ethics Committee of the university and registering in the Clinical Trials System of the Ministry of Health and Medical Education, and acquiring the written consent of patients’ parents, 60 patients in classes I and II in the ASA Physical Status Classification System, ranging between 3 and 12 years in age, who were tonsillectomy candidates were studied.

Exclusion criteria included metabolic and endocrine diseases, coagulation disorders, mental challenge, developmental disorders, any drug allergy history, peritonsillar abscesses, hypertension, psychotic disorders, chronic pain, and receiving analgesics.

Having ensured that the patients were NPO prior to the surgery, we gave all patients pre-anesthetic five minutes before administering the anesthetic. Anesthesia was performed identically in all patients (fentanyl 1 µg/1kg, sodium thiopental 5 mg/kg and atracurium 0.5 mg/kg). Prior to anesthesia, duringthe operation and in the recovery room, the peripheral arterial blood pressure, the PR and SpO2 were recorded. An anesthesiology assistant (BSc) prepared the 2 ml normal saline and ketamine syringes with special coding.

After tracheal intubation and administering anesthesia prior to surgical incision, the peritonsillar injection of 2 ml of normal saline and ketamine, in the control and intervention groups respectively, was performed fan-wise in the tonsillar bed and around it, from the upper and lower poles of the tonsils, by a surgeon, and the operation started five minutes later. Hemostasis was performed with sutures in both groups. The blood pressure, the heartbeat, the heart rate, and SpO2 were recorded every ten minutes throughout the surgery. The durations of the anesthesia and the operation were recorded by an anesthesiology assistant.

The data was collected using a 3-part questionnaire. The first was for the personal and social information of the patients, the second was for the CHEOPS (pain assessment) and the self-report Oucher Scale, and the third was for the Wilson Sedation Scale, the scientific validity and consistency of which have been shown in several studies (2 and 4).

The Oucher Scale is a self-report tool to measure pain in children aged 3-12 years old. The scale, which is used by all children’s healthcare personnel across the globe and can be accessed at oucher.org. Moreover, the content and structural consistencies of this scale have been documented using statistical tests. This scale is one of the most valid, oldest and most commonly used self-report pain scales in children, which was devised by Beyer. The scale consists of 6 child portraits showing different levels of pain, arranged vertically, from the lowest level at the bottom to the highest at the top, numbered 1 to 6 (1 for pain-free, and 6 the most severe pain). 

Pain assessment was performed by an anesthesiology assistant after the patient left the recovery room, assisted by the patient’s company.

The first part of the questionnaire was completed upon the patient’s entry to the OR, after extubation and the patient’s transfer to the recovery, and pain assessment using the CHEOPS and the Oucher Scale, sedation assessment using the Wilson Sedation Scale, and the assessment of complications were performed by an anesthesiology technician (MSc). 

Postoperative pain level assessments, the Wilson Sedation Scale and complications assessment were performed 5', 15', 30', 1 hour, 2 hours and 4 hours after the surgery, and the scores and the average score were recorded.

If the CHEOPS showed a pain score of 5+, acetaminophen was administered for pain relief. If other complications were present, proper treatments were performed.

All the questionnaire data were coded and recorded into SPSS. Then, the average Oucher Scale, CHEOPS and Wilson Sedation Scale scores were calculated. To compare the difference between the pain levels in the two groups, the t-test and the chi squared test were used, and to compare the sedation state, the Fischer’s exact test was used.

## Results

The results showed no significant difference between the intervention and the control groups in terms of sex, age, weight, height, and the average operation, anesthesia and extubation times (*P*>0.05) (Table 1).

**Table 1:** Patients’ demographics in the intervention and control groups

**Demographic variables**	**Ketamine group**	**Placebo group**	***P***
Age	2.51±7.93	2.47±7.40	0.219
Sex (Male/Female)	18.12	20.10	0.395
Weight	9.9±25.46	8.89±23.2	0.569
Height	13.43±126.63	22.65±119.38	0.435
Anesthesia time	7.85±34.66	7.92±35.43	0.660
Operation time	5.04±23	5.15±23.53	0.459
Extubation time	2.60±4.53	2.96±5.43	0.217

There was no significant difference in sleep patterns between the two groups on the first night after the surgery. Also, no complications, such as apnea and laryngospasm, were seen during the surgery or after the extubation in either group. 

There was also no significant difference in the time of discharge from the recovery room or the bed between the two groups. Respiration and hemodynamic patterns were identical in both groups. There was no significant difference in pain levels between males and females (*P*>0.05). 

Six point 67 percent of the control group and 3.33% of the ketamine group suffered from nausea during recovery, which was not a significant difference. Only 3.33% of the control group had postoperative hemorrhage and 1.33% of the ketamine group needed postoperative suction.

**Table 2:** Postoperative pain levels according to Oucher Scale

	**5 mins**	**15 mins**	**30 mins**	**60 mins**	**120 mins**	**240 mins**
**Ketamine**	0.531±1.17	1.155±1.67	1.177±1.83	0.994±1.67	0.828±1.22	0±1
**Placebo**	0.847±1.20	1.388±1.93	1.408±1.83	0.774±1.43	0.610±1.29	0.484±1.20
***P***	0.516	0.875	0.837	0.868	0.032	0.551

**Table 3:** Postoperative pain levels according to CHEOPS

	**5 mins**	**15 mins**	**30 mins**	**60 mins**	**120 mins**	**240 mins**
**Ketamine**	1.003±1.4	2.176±2.77	2.240±2.53	1.124±1.67	0.844±1.33	0.669±1.37
**Placebo**	1.073±1.53	1.810±2.63	1.807±2.67	1.213±1.9	1.358±1.53	0.711±1.33
***P***	0.577	0.726	0.971	0.769	0.057	0.798

**Table 4:** Postoperative sedation levels in the intervention and control groups according to the Wilson Sedation Scale

	**5 mins**	**15 mins**	**30 mins**	**60 mins**
**Ketamine**	0.85±3.37	0.900±2.13	0.568±1.23	0.0±1
**Placebo**	0.85±3.40	0.860±1.78	0.379±1.17	0±1
***P***	0.433	0.407	0.412	0.342
				

The ketamine group had a lower pain score compared with the control group (1.40±1.003 compared with 1.53±1.074). The average pain level on the Oucher Scale 2 hours after tonsillectomy was 1.22±0.828 in the ketamine group and 1.29±0.610 in the control group, which was a significant difference (*P*<0.032). The pain level on the CHEOPS in the ketamine group was also lower than that of the control group, which was a significant Difference (1.33±0.844 compared with 1.53±1.358) (Table 3).

16 point 67 percent of the ketamine group and 23.33% of the control group needed analgesic in the recovery room, but the difference was not significant (*P*<0.374). Only in 1.33% of the ketamine group was delirium seen in the recovery room. In neither group did patients need readmission after discharge.

## Discussion

The results of this study showed that pain levels experienced a falling trend on both the Oucher Scale and CHEOPS after the operation. On the CHEOPS, the average pain level 120 minutes after the surgery was lower in the intervention group than in the placebo group. On the Oucher Scale, the average pain level 120 minutes after the surgery was also lower in the ketamine group than in the control group.

Safavi et al also showed in their study that low-dose single peritonsillar injection of ketamine postponed the need for painkillers postoperatively and caused significant pain relief effects on the first day after the surgery (4). Dal et al also showed that peritonsillar use of ketamine reduced postoperative pain without any complications (2). The results of certain other studies have also shown low quantities of ketamine in outpatient surgeries reduce the perioperative pain and the need for analgesic administration by 35-40% (8).

Certain studies have shown that the positive effects of ketamine have persisted a few days after the surgery. Persistent effects of ketamine are caused by its effect on reducing secondary hyperalgesia and/or on preventing the spread of central sensitivity to peripheral pain (9).

There were no postoperative cases of apnea or laryngospasm in the ketamine group. Ketamine does not restrain the swallowing or pharyngeal reflexes, and stimulates the respiratory system by increasing the respiratory volume and rate of respiration (10).

Studies have shown that the preoperative administration of an NMDA receptor antagonist reduces the swallowing pain in adults after tonsillectomy (11).

In a large scale study on 1026 patients, pain levels in the ketamine group were shown to be lower than in the control group and patient satisfaction to be more (12). In some clinical studies, especially in visceral surgeries, ketamine has been seen to cause no pain relief (9). There was no difference in the levels of sedation according to the Wilson Sedation Scale at different times of the first hour, which was in accordance with the findings of the study conducted by Martindale et al (2004) (13).

Acute administration of narcotics may lead to delayed hyperalgesia which in turn results in the hyperactivity of NMDA receptors, activated by the narcotic stimulation, which may cause severe, acute pain. By blocking NMDA receptors, ketamine restrains this hyperalgesia and increases the duration and intensity of the pain relief induced by the narcotic (9).

Low doses of ketamine do not delay regaining consciousness; therefore, low-dose IV administration of ketamine in children undergoing tonsillectomy under general anesthesia reduces the postoperative pain (9).

The control group experienced more vomiting in the recovery room, but the difference was not significant. Nausea and vomiting were not major problems in the intervention and control groups, either (13), while PONV (postoperative nausea and vomiting) is one of the most common post-tonsillectomy problems (14). 

The need for analgesics was lower in the ketamine group than in the control group, but the difference was not significant. In the study by Marzban et al, the time of the first administration of the analgesic was longer in the ketamine group than in the control group, and the control group needed more peptide analgesics (9).

The limitations of this study were pain assessment in children and that the follow-up period spanned only the first 24 hours. In this study, the quantity and the dose of the ketamine injected in the tonsils were lower and slower than those injected IV. Therefore, it did not have sedative effects and the nausea and vomiting were less. We could not measure and study the serum levels of ketamine and norketamine after the peritonsillar injection; thus, we cannot say with certainty that the analgesic effects of this drug two hours later were due to its systemic absorption. The cultural differences among the patients may have also influenced the results of the study.

## Conclusion

Low-dose peritonsillar injection of ketamine before tonsillectomy reduces the swallowing pain and postoperative pain in children. The most likely explanation for the pain relief following the peritonsillar injection of ketamine is desensitization of the central nervous system to pain impulses. It is suggested that the onset and quality of oral feeding be investigated in future studies.
